# Enhancing Toxicology Achievement by the VARK and the GRSLSS-mixed Models in Team-Based Learning

**DOI:** 10.3389/fpubh.2021.732550

**Published:** 2022-01-18

**Authors:** Tanaporn Khamphaya, Phisit Pouyfung, Supabhorn Yimthiang

**Affiliations:** Occupational Health and Safety, School of Public Health, Walailak University, Nakhon Si Thammarat, Thailand

**Keywords:** toxicology education, learning style, team-based learning, VARK, GRSLSS

## Abstract

Toxicology is needed to implement in the occupational health and safety (OHS) curriculum. Teaching toxicology is very challenging as its multidisciplinary science. Keeping students engaged in learning is a difficult issue when introducing solely theoretical framework. To enhance student performance, educators need to be aware of different learning styles and teach students accordingly. This study aimed to examine preferred learning styles and to further investigate the impact of learning style on team allocation and the effectiveness of team-based learning (TBL) in toxicology. A cross-sectional study of OHS students was performed. The visual, aural, reading/writing, and kinesthetic (VARK) learning style questionnaire and the Grasha–Reichmann Student Learning Styles Scale (GRSLSS), which identifies independent, dependent, collaborative, participant, competitive, and avoidant learning styles, were used with 101 study participants. After classification, participants studied three aspects of toxicology in three respective situations: (i) individual learning, (ii) TBL with students of the same VARK learning style, and (iii) TBL with students of varying VARK learning styles. Afterward, participants wrote a test on each of the aspects. The dominant VARK and GRSLSS learning styles were reading/writing (33.33%) and collaborative (50.00%), respectively. The participants achieved the highest test scores (88.31%) when they studied in a team with the various VARK styles, followed by studying in a team with the same VARK style (83.43%). Individual learning produced the lowest average score (69.79%). The results of this study suggest that creating a successful heterogeneity team based on the preferred learning styles is an effective teaching method in toxicology. It might be useful to toxicology educators and research studies from a wide range of disciplines to enhance student performance.

## Introduction

Toxicology is one of the essential medical subjects used in occupational health and safety (OHS) in order to assess the hazard and risk of chemical and biological in the working environment. Toxicology content for OHS includes the topic of the principle of toxicology related to occupational health, the major class of industrial compounds, toxicant-related health effects by organ system, mechanism of toxicity, the use of biomarkers in clinical evaluation, clinical case studies for acute and chronic effects, toxicology and occupational medicine databases, and the prevention of adverse health effects. Students from various backgrounds are a very difficult task for toxicology teaching, especially when introducing biochemical concepts. Improving learning strategy of student is important for them to achieve the course learning outcome.

Team-based learning (TBL) is one of the active learning strategies that allowed students to work in a small group format (3–5 students/group). It has been reported that TBL is increasingly applied in several courses across medical curricula such as basic sciences, neurology, pharmacology, anatomy, pathology, and physiology (1, 2). TBL possesses the advantages of the student learning and social or group interactions (3). TBL focuses on brainstorming and discussion, resulting in problem-solving and knowledge application in a collaborative environment. The process increases the motivation for learning and creates a concept map that would lead to improve their recognition, application, and deep learning (4–7). Several publications reported that TBL is a very well-received and effective format in health professions education (8). However, it is not enough to only form a group and tell them to work together (9). The instructor must work to establish a social bond among members and/or group them to work together as a team.

The goal of team learning is to apply in-class knowledge to real-world situations by using simulation and question-guided learning. However, not all collaboration is fruitful. The previous study suggested that learning styles theories are useful for involving students in a variety of collaborative activities and providing supportive group facilitation (10–12). Thus, to create a more efficient curriculum, educators need to understand the learning styles of student in order to facilitate their learning (13). Learning style is a preferred way of processing and acquiring new knowledge of an individual (14, 15). Several studies have shown that matching learning styles of students with a curriculum or teaching style has improved test scores, while a mismatch has led to low academic achievement (16). Therefore, identifying learning styles and behaviors of students is valuable when developing a program that will produce efficient learners (13).

Among the growing number of tools used to determine learning preferences is one designed by Fleming and Mills (17), the visual, aural, read/write, and kinesthetic (VARK) questionnaire. The VARK questionnaire provides a greater understanding of the information processing of each student by reflecting the way students learn (18). The VARK has four categories, i.e., (i) visual (V): learners who best internalize and synthesize information when it is presented to them in a graphic depiction, diagrams, pictures, or colored word accents; (ii) auditory (A): learners who are most successful when they are allowed to hear information presented to them vocally; (iii) reading/writing (R): learners who work best in the reading/writing modality and demonstrate a strong learning preference for the written word, word lists, and text-based handouts; and (iv) kinesthetic (K): learners who need to take a physically active role in the learning process (19). In the recent years, the VARK has been used in many countries to assess the learning style preferences of several groups of undergraduate students of physiotherapy, nursing, dentistry, and allied health-care professional programs (20–25). In reviewing, most of them preferred the read/write and kinesthetic learning styles (23, 26).

The VARK is not a complete learning style inventory. It only provides users with a simple profile of their sensory learning preferences (27) and does not consider other learning criteria that are important in the science classroom, such as how students interact with peers and the teacher (28). The Grasha–Reichmann Student Learning Styles Scale (GRSLSS) is one of the few instruments designed to assess social interaction (29). The Grasha–Reichmann model focuses on: (i) student attitudes toward learning, classroom activities, teachers, and peers (30); and on (ii) how personal attributes influence learning strategies (31). The GRSLSS classifies the learning styles of student into six categories including: (i) avoidant: not enthusiastic about learning content and attending class; (ii) dependent: show little intellectual curiosity and learn only what is required; (iii) participant: good students in class; (iv) independent: students who like to think for themselves and are confident in their learning abilities; (v) competitive: students who learn the material in order to perform better than others in the class; and (vi) collaborative: typical of students who feel that they can learn by sharing ideas and talents (32). The GRSLSS has been studied across a variety of educational settings and among students in health professions such as pharmacy and medicine (33–37). However, there have been no recent studies of the social interaction model in OHS students. Therefore, an in-depth understanding of learning styles and behavior assessment using both the VARK and the GRSLSS would help the teacher to improve the performance of students.

As mentioned above, to improve the learning of toxicology in OHS students, the best possible learning environment, learning styles, and behavior should also be considered (3). TBL is one strategy that could improve the learning of OHS students. The previous study indicated that learning style preferences are valuable for engaging learners in collaborative learning of TBL (11). However, the effectiveness of TBL for students with various preferred learning styles is limited. Therefore, this study aimed to identify the learning styles of students using the VARK and the GRSLSS instruments and to further investigate the impact of learning style on team allocation and the effectiveness of TBL in toxicology. We then hypothesized that team allocation based on the learning styles of students could improve their TBL outcome compared to individual learning.

## Article Types

Curriculum, instruction, and pedagogy.

## Manuscript Formatting

### Methodology

#### Student Population

The study type was cross-sectional. It was conducted at the Department of OHS, School of Public Health, Walailak University, Thailand, from November 2019 to February 2020. The students who were registered and attended the toxicology class in the second semester of the 2019 academic year were invited to participate in this study. In this study, a population is an entire group of students who enrolled in the toxicology class, which consists of 101 students. All the students who provided informed written consent to participate in this study were included (*n* = 101). Inclusion criteria were as follows: (i) age ≥ 18 years; (ii) attend all the topics of this course; and (iii) ability to read, write, and understand the Thai language. Exclusion criteria were as follows: (i) unwilling to participate in this study or (ii) not possible to be contacted during the data collection period.

#### Study Instruments

To determine the instructional preferences and social interaction models learning styles, the VARK and the GRSLSS were used because they are reliable, concise, and easy to complete. They have also been used widely in the field of education among health profession students (21, 38, 39). The latest Thai version of the VARK questionnaire (version 8.01) was sourced online (https://vark-learn.com/). It consists of 16 questions with 4 options each and the respondents could select more than one response for each question if deemed suitable. In addition, the Thai version of the GRSLSS was adapted from Visudtibhan and Disorntatiwat (40). The GRSLSS is a five-point Likert-type scale consisting of 60 items with six subscales (independent, dependent, avoidant, participative, competitive, and collaborative) to identify the learning styles of the students (29). Demographic data (age and gender) and educational background comprised the first section of the questionnaire, followed by the VARK and the GRSLSS, which had been approved and validated by many studies (38, 39, 41).

#### Data Collection

The questionnaires were distributed in the form of hard copies to students in the classroom on the first day of class. After obtaining informed written consent, the participants were invited to respond to the questionnaires anonymously. The students were given 60 min to complete the questionnaires. If a student was found to be absent on occasions, he/she was excluded from this study. The learning styles of the students were identified according to the VARK and the GRSLSS and they were categorized according to their predominant (the highest scoring) styles.

#### Team-Based and Individual Learning

Students were taught three topics for 45 min on each topic, using traditional lectures and materials such as PowerPoint slides, online videos, and selected textbooks. For group allocation, all the students were randomly grouped based on the VARK and the GRSLSS styles according to Table 1. After that, all the students were assigned to learn by: (i) themselves (individual learning) and by means of TBL, which was divided into two subgroups, i.e., (ii) TBL with the same VARK style in the same group, and (iii) TBL with the mixed VARK styles in each group (Table 1). Learning objectives were announced at the beginning of each topic (Table 1). Students studied for 1 h on each topic, with class and outsource materials and question guidelines using the Socrative approach, to check their understanding and give them feedback in real time. After each topic, students were also tested by Socrative application and the test scores were collected.

**Table 1:** Detail of individual and team-based learning experiment.

**Type of learning**	**Number of students**	**Group allocation method**		**Topic**	**Learning objectives**	**Learning activities**	**Resources**
		**VARK**	**GRSLSS**				
Individual	101	–	–	Metal toxicity	Describe the chemical mechanism of metal toxicity, and biomarkers of metal exposure.	Traditional lecture 45 *min*, individual learning using questioning technique 60 *min*, real time feedback	Powerpoint slides, online videos, textbooks
TBL1	101 (5–6 students/group)	Randomly group by V, A, R, K, or multimodal	Randomly group by GRSLSS (2 collaborative students/group)	Toxic effects of solvents and vapors	Explain and describe the chemical mechanism of solvent and vapor toxicity, and biomarkers of solvent and vapor exposure.	Traditional lecture 45 *min*, TBL using questioning technique 60 *min*, real time feedback	
TBL2	101 (5–6 students/group)	Randomly group by VARK (Each group contains variety of V, A, R, and K)	Randomly group by GRSLSS (2 collaborative students/group)	Environmental toxicity (water, soil, air pollutants)	1. Describe and identify type and source of pollutants in environment (water, soil, and air pollutants) 2. Explain and describe the mechanism of toxicity and biomarker of major pollutant from water, soil and air.	Traditional lecture 45 *min*, TBL using questioning technique 60 *min*, real time feedback	

*Under line means students were not allocated into group because of individual learning*.

### Ethical Considerations

This study protocol was approved by the Institutional Review Board on the Protection of the Rights of Human Subjects (Walailak University), which was conducted in accordance with the Declaration of Helsinki. The topics, teaching materials, and questionnaire guidelines to evaluate learning outcomes were approved by Walailak University committees. The Human Investigation Committee protocol number is WUEC-19-211-01. All the students responded anonymously to the study questionnaire.

### Statistical Analysis

Data generated from the VARK and the GRSLSS questionnaire were analyzed according to the method described previously (19, 41). Results were presented in the form of descriptive statistics and all data were analyzed by the GraphPad Prism version 7 Software (GraphPad, La Jolla, California, USA). Data are presented as means ± SEM. Statistical analyses were performed using the two-tailed unpaired Student's *t*-test or one-way ANOVA when three or more groups were compared. Differences were considered statistically significant at *p* < 0.05.

## Results

### Demographic Data

The total number of participants was 101 students (males *n* = 12 and females *n* = 89) and the response rate was 100%. The mean age, the accumulated grade point average (GPAX), and toxicology grade of students were 20.79 ± 0.09, 2.53 ± 0.04, and 3.07 ± 0.07, respectively (Table 2).

**Table 2:** Baseline demographic data.

**Variable**	**Data**
Number of students	101
Male	12
Female	89
Age (mean ± S.E.M.)	20.79 ± 0.09
GPAX	2.53 ± 0.04
Toxicology Grade	3.07 ± 0.07

### Visual, Aural, Reading/Writing, and Kinesthetic Learning Style

**Figure 1:**
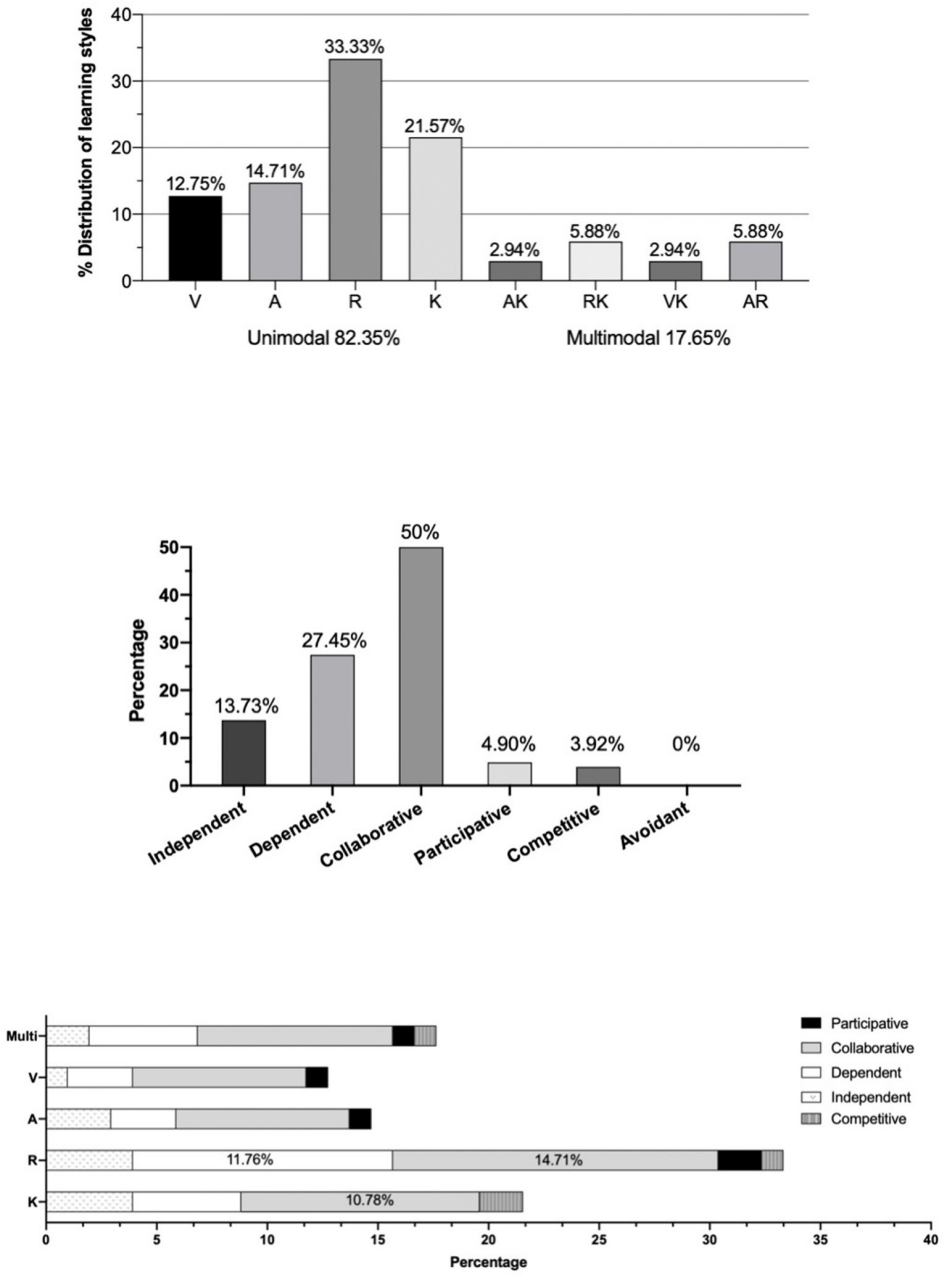
The Visual, aural, read/write, and kinesthetic (VARK) and the Grasha–Reichmann Student Learning Styles Scale (GRSLSS) learning styles.

The learning styles of students were categorized into unimodal (82.35%) and multimodal (17.65%) patterns. Within the unimodal pattern, the dominant learning style was reading/writing (33.33%), followed by kinesthetic (21.57%), aural (14.71%), and the least presented was visual (12.75%). All the multimodal patterns were bimodal. Among the multimodal patterns, the dominant combinations were RA and RK (5.88%) followed by KV and KA (2.94%) (Figure 1; Supplementary Table 1).

### Grasha–Reichmann Student Learning Styles Scale Learning Style

The dominant social learning style according to the GRSLSS was collaborative (50%), followed by dependent (27.45%), independent (13.63%), competitive (4.90%), and participative (3.92%), with no participant having the avoidant style as the dominant one (Figure 1). The GRSLSS subscale scores are given in Table 3. The highest subscale score was also collaborative, while the lowest score was competitive.

**Table 3:** The Grasha–Reichmann Student Learning Styles Scale (GRSLSS) subscale scores.

***n* = 101**	**Mean ±S.E.M**.	**Minimum**	**Maximum**
Independent	3.91 ± 0.03	3.00	4.6
Dependent	3.54 ± 0.04	2.60	4.8
Collaborative	3.93 ± 0.06	2.10	5.0
Participative	3.51 ± 0.04	2.20	4.8
Competitive	2.85 ± 0.06	1.40	4.6
Avoidant	2.87 ± 0.05	1.70	4.1

### Mixed Models and TBL Results

**Figure 2:**
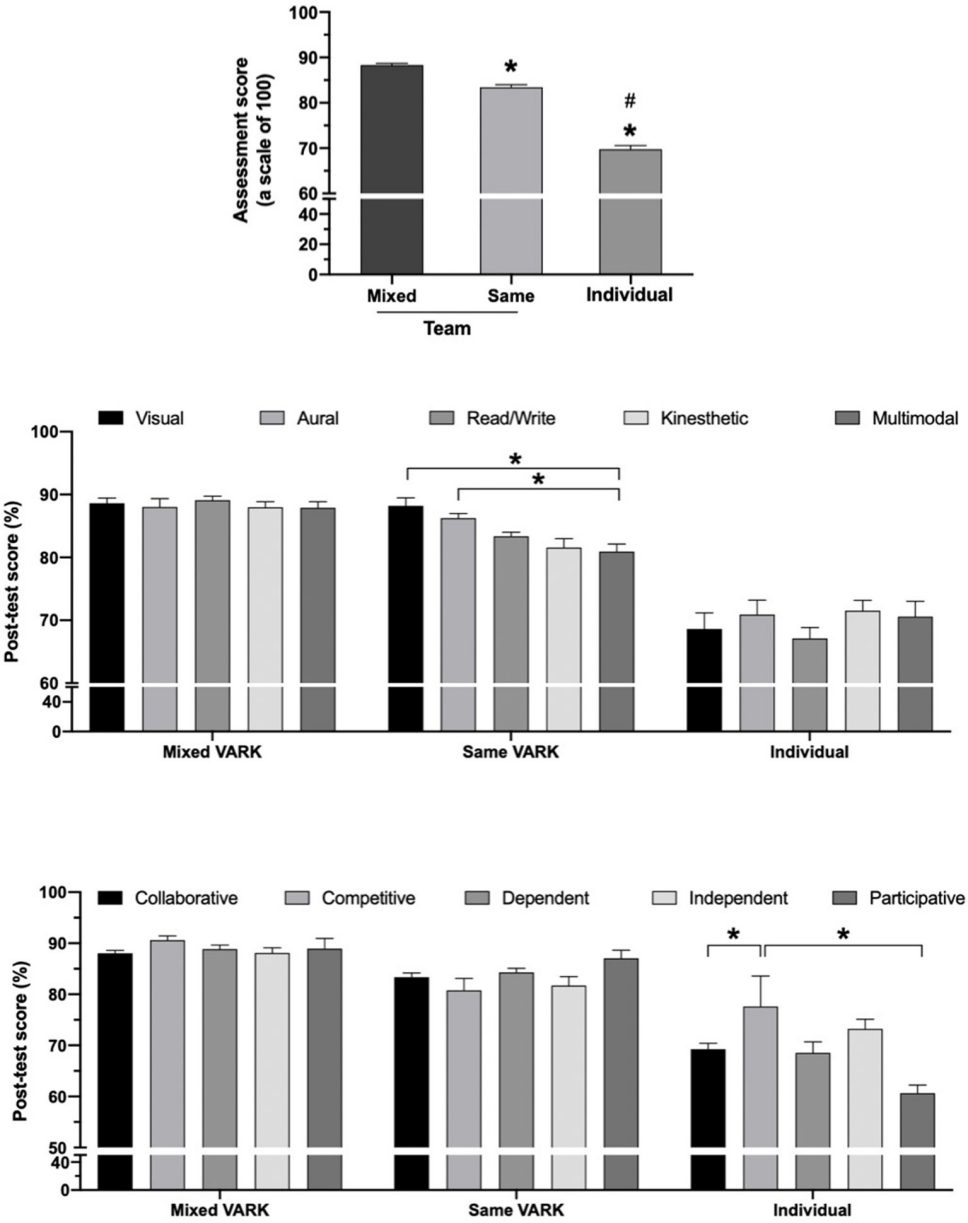
Mixed VARK and team-based learning (TBL) results. ^*^p < 0.05 compared to mixed VARK–TBL and ^**#**^*p* <0.05 compared to same VARK–TBL (*top panel*) and ^*^p < 0.05 between two groups (middle and bottom panels).

Since 50% of the participants had a collaborative learning style, we then hypothesized that TBL would enhance student performance. To confirm whether the participants preferred to share ideas with other students and teachers and could achieve better scores when working as a team, the three study topics were used and students were tested after team-based and individual learning (Table 1). We found that students scored the highest (88.31%) when they did TBL with various VARK styles in a team (5–6 students), followed by TBL with the same VARK style in a team (83.43%). However, the scores were significantly lower when they learned individually (69.79%) (Figure 2). It suggests that studying as a team with the different VARK learning styles enhanced the test scores of students.

## Discussion

Teaching style plays an important part in letting students engage with their knowledge, which could increase student performance and understanding. Therefore, teachers need to develop the best teaching methods and materials for their students. An understanding of the learning styles of student is one of the strategies for developing teaching materials suitable for individual students. The results in this study revealed that the most preferred learning styles among OHS students were reading/writing (R) in terms of the VARK and collaborative learning in terms of the GRSLSS. In addition, TBL consisting of students with mixed modality and collaborative preferences produced better toxicology learning performance than individual learning.

Niel Fleming divides instructional preferences of students into the four types of the VARK according to how they best acquire information (19). However, there can also be a multimodal classification when a student has two to four learning styles (22, 42). The majority of participants in this study were unimodal (82%), and the predominant style was read/write. This result is consistent with those of other studies on health profession students, such as medical students from Indiana, USA (43), and nursing students from Thailand (44). However, medical students from Kuala Lumpur preferred the kinesthetic style (45), the second most common learning style in this study (Figure 1). According to other previous research, there are various learning styles among health profession students. For example, dental students in Philadelphia, USA, and medical students in Saudi Arabia mostly had visual and auditory learning styles, respectively (22, 42). These variances in the learning styles across countries could be due to cultural differences and the levels of experience of the students (45).

Some students could have multimodal learning styles. For example, more than 60% of caregivers from the USA (46) and medical students from the USA (43), Turkey (47), Saudi Arabia (42), Iran (48), and China (49) were multimodal learners. However, only a small proportion was found in our OHS students, which was similar to undergraduate medical students in Kuala Lumpur (45). It could be due to (i) the difference in teaching instruction from their previous year or how they studied when they were in high school (45) and (ii) their experience, as the multimodal learning styles are usually found in people aged between 20 and 29 years (45).

The VARK provides users only a simple profile of their sensory learning preference (27) and does not consider other learning criteria such as how students interact with peers and the teacher (28). In the present day, there is a perceived shift from teacher-centered or traditional to student-centered learning, in which students learn and work together in their groups. In addition, the OHS program has a combined active learning and traditional style curriculum. Our goal in the OHS discipline is teaching that is mainly based on practical training and active participation in group work, developing learners to acquire the learning skills required in the 21st century. To design a more efficient curriculum and student study group activity, educators need to understand both how students acquire new knowledge and how they interact in the classroom. The OHS students in this study were assigned to work with their peers, and it is fortunate that the most preferred social interaction model was collaborative (50%), which was similar to Turkish physiotherapy students (38%) (21), medical students from Indian Medical College (50), and pharmacy students (36). Follow by the second most learning style found in OHS, dependent, which is also similar to Indian medical students (50). This type of student sees teachers as the authority and learns only what is created by the teacher.

Several studies have suggested that the instructor needs (i) to understand their learning style of students, (ii) to select educational activities and match with learning style preferences, and (iii) to value the diversity of the learning styles within the group. To avoid a dysfunctional group, we then allocated the team by using the VARK and the GRSLSS learning styles. Free riding or social loafing is the main factor of unsatisfactory group-work experiences (51). In addition, individual characteristics, such as psychological profile and learning preferences, may influence team performance (52). We then assigned at least 2 collaborative students in each team and, as expected, average scores of students were higher when they worked together (53, 54). Our result suggests that this TBL could be fitted with the collaborative learning style classified by the GRSLSS for several reasons. First, collaborative students can learn better with a collaborative instructional environment (11). Second, not only the collaborative student but dependent and participant students were also compatible with these TBL learning methods (11). However, when dependent/collaborative students learned individually, their performances were lower than the competitive learners (Figure 2). It is clear that collaborative/dependent/participant students learn better when they were coached by their instructor and surrounded by their peers. Third, students with TBL get a higher score could be due to TBL methodology itself. The teachers gave them the main and subtopics in each section and allowed them to spend more time discussing and sharing ideas with their teachers and peers and to ask about what they did not understand. This strategy may facilitate collaborative learning to improve a sharing environment in the classroom. Therefore, organizing the curriculum to include small group discussions may be beneficial and could help students to get more information and higher scores than individual learning.

As we mentioned above, TBL improved learning scores when compared to individuals (Figure 2). Interestingly, average score of mixed VARK–TBL was higher than the same VARK–TBL (Figure 2, *middle panel*). This could be due to students tend to learn better when they are involved in various VARK-learning style participation (12). However, a significant change of *post-test* score was observed within the same VARK-TBL, especially when compared between V/A and multimodal. We found that *post-test* scores of multimodal students were lower than those of students who only studied with their visual or aural peers (Figure 2, middle panel). It could be because V and A can remember and get information by watching and listening faster than others (55). However, these results were inconsistent with previous reports that multi-VARK models can adjust to the different teaching styles and lead to higher scores from active learning strategies (39, 56). This could be due to the different multimodal groups between the two studies. Surprisingly, a competitive style could help students to get higher scores when they learned individually, indicating that they were likely to compete with other students in the class to get a higher grade (Figure 2; Supplementary Figure 1). In contrast, previous studies found that the participative style usually leads to higher academic performance than others when students enjoy and have an interest in in-class activities (21). Therefore, less activity and discussions during individual learning might cause them to be bored or inattentive, and their score would be lower. This may suggest that increasing in-class activities and group discussions with various VARK styles in one group would be beneficial for students.

For each test, TBL helped students to get a higher score when compared with individual learning. It seems that TBL could help students improve their scores and performances in the class (57). However, our students did not show significantly different scores in their Toxicology grade and GPAX (Supplementary Figure 1 and Supplementary Tables 2 and 3). There was no evidence that any particular learning style from the VARK and the GRSLSS was superior to others (Supplementary Figure 1) (15, 26, 58, 59). It could be because: (i) our teaching method and material might be suitable for all students but not specific to each learning style and (ii) students were not aware of their learning styles and were not exposed to collaborative learning by forming a group and studying together before the final examinations. Several studies found that an awareness of one's own learning style could greatly enhance success in the summative examination (45, 60–63). However, the average toxicology grade of student ranged from 3.0 to 3.2 (Supplementary Table 2; Supplementary Figure 1), which was acceptable as a good level when compared to the last toxicology course (unpublished data). It could be due to the feedback that all teachers gave their students right away after the postexperiment test. Therefore, all the types of learners would have equal information and get the same score (Supplementary Figure 1).

To the best of our knowledge, this is the first study that used both the VARK and the GRSLSS learning styles in toxicology teaching among OHS students in Thailand higher education. However, this study has some limitations. First, the learning style can be changed based on the experience of student (21). It should be kept in mind that the conclusions of this study could be limited due to the cross-sectional design. Therefore, the prospective study design should be considered for further study. The second limitation of this study is the low number of students. Finally, the sample was from a single University and not representative of all the OHS students in Thailand. A larger study from multiple universities is needed in the future.

## Conclusion

In conclusion, this study demonstrated that OHS students prefer to learn using read/write with a collaborative learning style. They adopted both the modality (VARK) and team learning (GRSLSS) preference in group allocation that indicated better performance when compared to individual learning. In summary, this study guides creating a successful heterogeneity team based on the preferred learning styles. This information is very useful for improving the quality of teaching and may impact how educators create the toxicology classroom environment.

## Data Availability Statement

The original contributions presented in the study are included in the article/Supplementary Material, further inquiries can be directed to the corresponding author/s.

## Ethics Statement

The studies involving human participants were reviewed and approved by the Institutional Review Board on the Protection of the Rights of Human Subjects, Walailak University (WUEC-19-211-01). The patients/participants provided their written informed consent to participate in this study.

## Author Contributions

TK, PP, and SY designed and performed study. TK analyzed data and drafted the manuscript. PP and SY reviewed and edited manuscript. SY supervised the research. TK, PP, and SY contributed to the article and approved the submitted version.

## Funding

This study received no specific grant from any funding agency in the public, commercial, or not-for profit sectors.

## Conflict of Interest

The authors declare that the research was conducted in the absence of any commercial or financial relationships that could be construed as a potential conflict of interest.

## Publisher's Note

All claims expressed in this article are solely those of the authors and do not necessarily represent those of their affiliated organizations, or those of the publisher, the editors and the reviewers. Any product that may be evaluated in this article, or claim that may be made by its manufacturer, is not guaranteed or endorsed by the publisher.
