# Supplementary material for: Enhancing Toxicology Achievement by the VARK and the GRSLSS-mixed Models in Team-Based Learning
Source: Front Public Health. 2022 Jan 18;9:732550. doi: 10.3389/fpubh.2021.732550 (PMC8804276; doi:10.3389/fpubh.2021.732550)
Supplement: Supplementary file 1 [file Data_Sheet_1.docx]

**SUPPLEMENTARY DATA**

**Supplementary Table 1**

*Percentage of VARK and GRSLSS Learning Styles among Occupational Health and Safety Students*

|  | Independent | Dependent | Collaborative | Participative | Competitive | Total |
| --- | --- | --- | --- | --- | --- | --- |
| V | 0.98 | 2.94 | 7.84 | 0.98 | 0.00 | 12.75 |
| A | 2.94 | 2.94 | 7.84 | 0.98 | 0.00 | 14.71 |
| R | 3.92 | 11.76 | 14.71 | 1.96 | 0.98 | 33.33 |
| K | 3.92 | 4.90 | 10.78 | 0.00 | 1.96 | 21.57 |
| Multi | 1.96 | 4.90 | 8.82 | 0.98 | 0.98 | 17.65 |
| Total | 13.73 | 27.45 | 50.00 | 4.90 | 3.92 |  |

**Supplementary Table 2**

*Toxicology Grade in VARK and GRSLSS Learning Styles*

|  | Independent | Dependent | Collaborative | Participative | Competitive | Average |
| --- | --- | --- | --- | --- | --- | --- |
| V | 3.00 ± 0.00 | 2.83 ± 0.73 | 3.31 ± 0.27 | 2.50 ± 0.00 |  | 2.91 ± 0.17 |
| A | 3.00 ± 0.29 | 3.00 ± 0.00 | 2.71 ± 0.31 | 4.00 ± 0.00 |  | 3.18 ± 0.28 |
| R | 3.00 ± 0.54 | 3.04 ± 0.25 | 3.20 ± 0.13 | 3.25 ± 0.75 | 3.50 ± 0.00 | 3.20 ± 0.09 |
| K | 3.25 ± 0.25 | 3.30 ± 0.20 | 3.04 ± 0.21 |  | 2.75 ± 1.25 | 3.02 ± 0.18 |
| Multimodal | 2.25 ± 0.25 | 3.00 ± 0.16 | 3.06 ± 0.24 | 3.00 ± 0.00 | 3.50 ± 0.00 | 2.96 ± 0.20 |
| Average | 2.90 ± 0.17 | 3.03 ± 0.08 | 3.06 ± 0.10 | 3.19 ± 0.31 | 3.17 ± 0.33 |  |

Note: Data represented as mean ± S.E.M.

**Supplementary Table 3**

*GPAX and VARK and GRSLSS Learning Styles*

|  | Independent | Dependent | Collaborative | Participative | Competitive | Average |
| --- | --- | --- | --- | --- | --- | --- |
| V | 2.81 ± 0.00 | 2.64 ± 0.28 | 2.58 ± 0.15 | 2.49 ± 0.00 |  | 2.63 ± 0.07 |
| A | 2.60 ± 0.10 | 2.46 ± 0.15 | 2.40 ± 0.16 | 3.06 ± 0.00 |  | 2.63 ± 0.15 |
| R | 2.77 ± 0.37 | 2.63 ± 0.12 | 2.64 ± 0.08 | 2.80 ± 0.43 | 2.67 ± 0.00 | 2.70 ± 0.03 |
| K | 2.73 ± 0.29 | 2.59 ± 0.08 | 2.63 ± 0.15 |  | 2.82 ± 0.26 | 2.69 ± 0.05 |
| Multimodal | 2.37 ± 0.13 | 2.57 ± 0.09 | 2.61 ± 0.10 | 2.84 ± 0.00 | 2.97 ± 0.00 | 2.67 ± 0.11 |
| Average | 2.66 ± 0.08 | 2.58 ± 0.03 | 2.57 ± 0.04 | 2.80 ± 0.12 | 2.82 ± 0.09 |  |

Note: Data represented as mean ± S.E.M

**Supplementary Table 4**

*Toxicology grades and number of students in each learning styles.*

|  | Visual | Aural | Read/Write | Kinestatics | Multi |
| --- | --- | --- | --- | --- | --- |
| Independent | B = 1 | B+ = 1 | A = 1 | A = 1 | B = 1 |
|  |  | B = 1 | B+ = 1 | B = 3 | C+ = 1 |
|  |  | C+ = 1 | D+ = 1 |  | C = 1 |
| Dependent | A = 1 | B = 3 | A = 4 | A = 1 | B+ = 1 |
|  | B = 1 |  | B+ = 1 | B+ = 1 | B = 3 |
|  | D+ = 1 |  | B = 2 | B = 3 | C+ = 1 |
|  |  |  | C+ = 3 |  |  |
|  |  |  | C = 1 |  |  |
|  |  |  | D+ = 1 |  |  |
| Collaborative | A = 3 | A = 1 | A = 1 | A = 2 | A = 2 |
|  | B+ = 2 | B+ = 1 | B+ = 7 | B+ = 3 | B+ = 2 |
|  | B = 1 | C+ = 4 | B = 5 | B = 1 | B = 1 |
|  | C+ = 1 | D+ = 1 | C+ = 1 | C+ = 4 | C+ = 3 |
|  | C = 1 |  | C = 1 | C = 1 | C = 1 |
| Participative | C+ = 1 | A = 1 | A = 1 |  | B = 1 |
|  |  |  | C+ = 1 |  |  |
| Competitive |  |  | B+ = 1 | A = 1 |  |
|  |  |  |  | D+ = 1 |  |

**
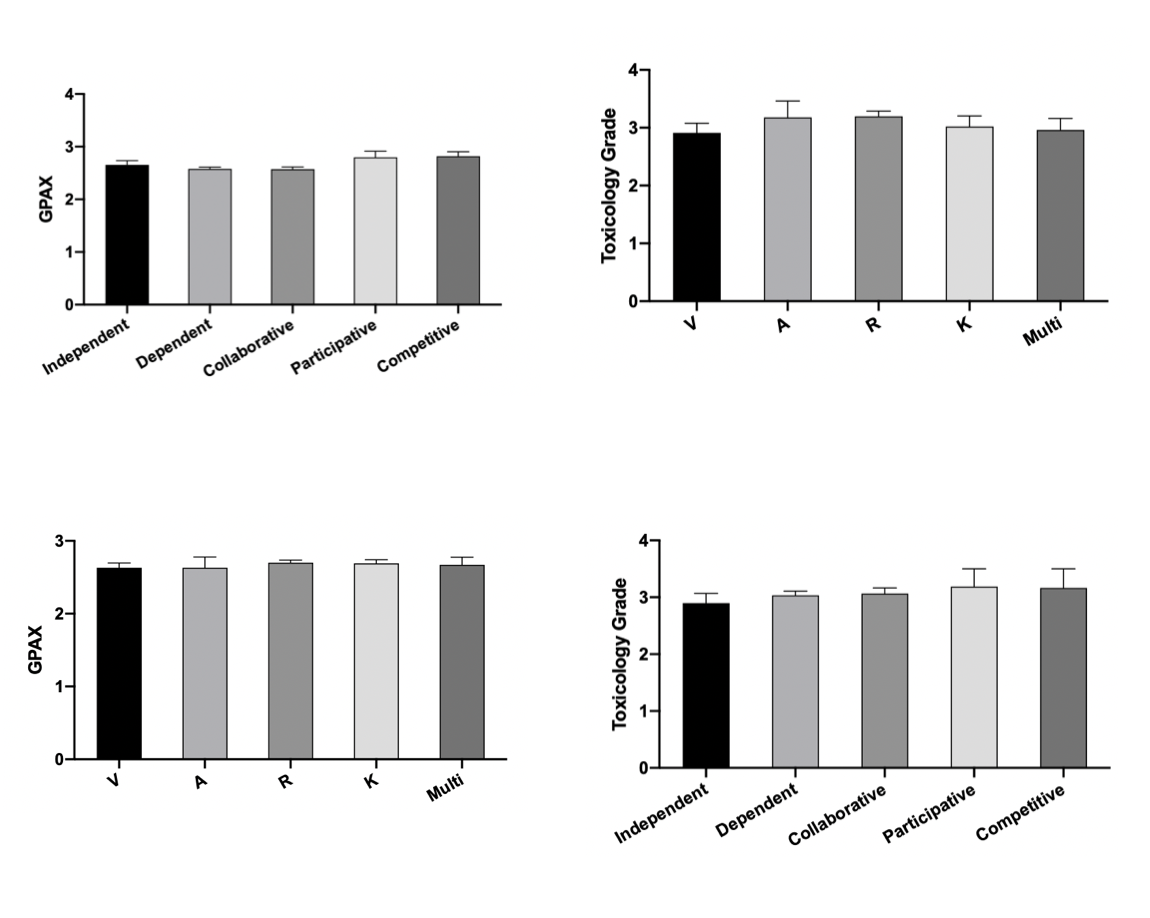
**

*Supplementary Figure 1.* VARK, GRSLSS, GPAX, and toxicology grades.
